# High frame rate doppler ultrasound bandwidth imaging for flow instability mapping

**DOI:** 10.1002/mp.13437

**Published:** 2019-03-04

**Authors:** Billy Y. S. Yiu, Adrian J. Y. Chee, Guo Tang, Wenbo Luo, Alfred C. H. Yu

**Affiliations:** ^1^ Schlegel Research Institute for Aging Department of Electrical & Computer Engineering University of Waterloo Waterloo ON N2L 3G1 Canada; ^2^ Bioprober Corporation Seattle WA 98004 USA

**Keywords:** autoregressive modeling, Doppler bandwidth estimation, flow instability, high frame rate ultrasound

## Abstract

**Purpose:**

Flow instability has been shown to contribute to the risk of future cardiovascular and cerebrovascular events. Nonetheless, it is challenging to noninvasively detect and identify flow instability in blood vessels. Here, we present a new framework called Doppler ultrasound bandwidth imaging (DUBI) that uses high‐frame‐rate ultrasound and Doppler bandwidth analysis principles to assess flow instability within an image view.

**Methods:**

Doppler ultrasound bandwidth imaging seeks to estimate the instantaneous Doppler bandwidth based on autoregressive modeling at every pixel position of data frames acquired from high‐frame‐rate plane wave pulsing. This new framework is founded upon the principle that flow instability naturally gives rise to a wide range of flow velocities over a sample volume, and such velocity range in turn yields a larger Doppler bandwidth estimate. The ability for DUBI to map unstable flow was first tested over a range of fluid flow conditions (ranging from laminar to turbulent) with a nozzle‐flow phantom. As a further demonstration, DUBI was applied to assess flow instability in healthy and stenosed carotid bifurcation phantoms.

**Results:**

Nozzle‐flow phantom results showed that DUBI can effectively detect and visualize the difference in Doppler bandwidth magnitude (increased from 2.1 to 5.2 kHz) at stable and unstable flow regions in an image view. Receiver operating characteristic analysis also showed that DUBI can achieve optimal sensitivity and specificity of 0.72 and 0.83, respectively. In the carotid phantom experiments, differences were observed in the spatiotemporal dynamics of Doppler bandwidth over a cardiac cycle. Specifically, as the degree of stenosis increased (from 50% to 75%), DUBI showed an increase in Doppler bandwidth magnitude from 1.4 kHz in the healthy bifurcation to 7.7 kHz at the jet tail located downstream from a 75% stenosis site, thereby indicating flow perturbation in the stenosed bifurcations.

**Conclusion:**

DUBI can detect unstable flow. This new technique can provide useful hemodynamic information that may aid clinical diagnosis of atherosclerosis.

## 1 Introduction

Risk stratification of carotid atherosclerotic stenosis is conventionally based on measurements of luminal narrowing^1^ and peak systolic velocity.^2^ However, atherosclerotic plaques with identical degree of stenosis could have substantial differences in their associated risk,^3^, ^4^ thereby prompting for diagnostic considerations beyond the immediate stenotic site.^5^ From a hemodynamics standpoint, the presence of stenosis perturbs blood flow, and in turn it may lead to flow instability.^6^ This phenomenon forms the underlying basis for physical examinations of carotid atherosclerosis via the detection of carotid bruit using a stethoscope.^7^ Unstable blood flow (i.e., nonlaminar flow, including turbulence) has also been associated with atherogenesis and plaque progression,^8^ as well as an increased risk for thrombosis and embolization.^9^, ^10^ Thus, monitoring blood flow instability could offer new clinical insights to understand the mechanism of atherosclerotic plaque development.^11^, ^12^

Instability of flow is characterized by the fluctuations of flow velocities in space and time.^13^ Medical imaging modalities have been leveraged to derive the flow turbulence index,^14^, ^15^, ^16^ a parameter describing the variance in flow velocities at specific cardiac instances between multiple consecutive cardiac cycles. For example, phase‐contrast magnetic resonance imaging has demonstrated feasibility in mapping turbulent flow regions from measurements of intra‐voxel mean velocity variations.^14^, ^17^ Similarly, Doppler ultrasound was used to calculate the turbulence index by measuring the standard deviation of flow velocities over successive cardiac cycles.^18^, ^19^ However, fluctuations in heart rate and stroke volume^20^ pose a significant drawback for the turbulence index approach due to its susceptibility to inter‐cardiac‐cycle variations.

Instead of relying on measurements over multiple cardiac cycles, it is possible to detect unstable flow by identifying high‐frequency velocity fluctuations at specific instants in a cardiac cycle.^21^ Instantaneous blood velocity fluctuation can be measured using an intravascular catheter,^22^ but this approach is highly invasive and is thus not ideal. As an alternative, Doppler ultrasound has been used to noninvasively detect flow instability in the form of Doppler spectral broadening (as an effect of flow velocity fluctuations) based on the characterization of Doppler spectral bandwidth.^23^, ^24^, ^25^ This approach has demonstrated initial success in assessing plaque risk.^26^ Yet, Doppler ultrasound, which only operates on a single range gate,^27^ is far from ideal in mapping flow instability because it lacks the ability to track fluctuations in flow velocities in multiple spatial positions.

Perhaps one workaround of Doppler ultrasound's single‐gate data acquisition paradigm is to perform Doppler‐based color flow imaging (CFI) that can provide color‐coded rendering of mean axial velocity estimates or velocity variance estimates.^28^ With CFI, which is essentially a full‐view version of single‐gate Doppler ultrasound, unstable flow regions may be visually identified as puff‐like color patches^29^ or mosaic color patterns^30^, ^31^ in the CFI frame. Nevertheless, there are multiple caveats in using CFI for flow instability analysis. First, although CFI can yield real‐time frame rates within the video display range (~20 fps), its time resolution is inadequate to follow the fast‐changing nature of unstable flow.^32^ Second, its data acquisition conventionally involves multiple firings over each of the scanlines in the image view,^28^ so each CFI frame is not able to capture a coherent spatial snapshot of unstable flow. Third, CFI's derivation of flow estimate at a pixel position is inherently prone to significant fluctuations and inaccuracies because each slow‐time ensemble used for Doppler processing is limited in size (8–16 samples) as constrained by real‐time requirements. Given all these issues, it is well possible that puff‐like or mosaic coloring patterns in a CFI frame are simply spurious artifacts rather than true indications of unstable flow, especially if CFI parameters are not tuned properly.

In this work, we present a new ultrasound framework called Doppler ultrasound bandwidth imaging (DUBI) that can map flow instability at all pixel positions within the image view based on local Doppler bandwidth analysis. In formulating DUBI, we hypothesized that broad‐field insonification methods, which are used for high‐frame‐rate ultrasound imaging,^33^ can be leveraged to: (a) facilitate simultaneous mapping of flow instability at all pixel positions, and (b) acquire slow‐time data with larger ensemble sizes than that obtainable in CFI and without lengthening the observation period, as required for consistent Doppler bandwidth analysis. We further hypothesized that to achieve consistent characterization of Doppler bandwidth at each pixel position, model‐based signal processing tools such as autoregressive (AR) modeling^34^ may be applied as they are known to yield consistent Doppler spectra estimates. Note that the merit of AR modeling in conventional Doppler ultrasound has previously been established.^35^, ^36^, ^37^ This technique can effectively reduce random fluctuations in the Doppler spectra and is less sensitive to intrinsic spectral broadening caused by the use of small Doppler window sizes.^36^, ^38^

This paper shall present the theoretical (Section 2.A) and implementation (Section 2.B) details of DUBI. Also, based on a nozzle‐flow model (Section 2.C), a receiver operating characteristic (ROC) analysis of DUBI's efficacy will be presented (Section 3.A) with reference to benchmark images derived from contrast‐enhanced ultrasound (CEUS).^39^ Further characterization of DUBI's performance will be presented in terms of its ability to identify flow instability in anthropomorphic carotid bifurcation phantoms with different degrees of stenosis (Section 3.B).

## 2 Materials and methods

### 2.A. Theoretical principles of DUBI

#### 2.A.1. Background description

Doppler ultrasound bandwidth imaging is founded upon the general principle that unstable flow can be characterized by the presence of a wide range of velocities (due to velocity fluctuation) at a given pixel position (or sample volume).^40^ Such extended range of velocities represents an extrinsic factor for Doppler spectral broadening^23^, ^24^, ^25^ and would result in a wider bandwidth. To track changes in Doppler bandwidths over all pixel positions, we have exploited the following principles:

1. Broad‐view insonation as enabled by unfocused plane wave transmission strategy^33^;
2. Coherent broad‐view image formation by parallel beamforming of channel‐domain pulse‐echo data received from each unfocused transmission^41^;
3. Simultaneous slow‐time sampling for all pixels within the image view^42^, ^43^;
4. AR modeling to derive instantaneous Doppler bandwidth at each pixel position.

In the following, further details will be presented on how each principle was incorporated into DUBI.

Note that, aside from velocity fluctuations due to unstable flow, two other factors may lead to extrinsic Doppler spectral broadening, including: (a) the existence of a spatial flow velocity gradient within the sample volume^23^, ^24^; (b) temporal variations in flow velocity (i.e., acceleration or deceleration).^36^ DUBI has sought to reduce the impact of these two alternative sources of extrinsic spectral broadening by limiting the effective axial range, lateral width, and observation period (to be discussed further in Section 2.B). Doppler spectral broadening may also arise intrinsically (sometimes referred to as Doppler ambiguity^23^) due to the finite transit time of blood scatterers^44^ and, to a lesser extent, flow instability.^23^ DUBI was developed on the assumption that transit‐time broadening was not as significant as extrinsic Doppler spectral broadening due to unstable flow. The intrinsic spectral broadening effects of flow instability were assumed to be insignificant too since it was confirmed to be even less prominent than transit‐time broadening.^24^

#### 2.A.2. Coherent slow‐time sampling of blood flow using broad‐view ultrasound imaging

**Figure 1:**
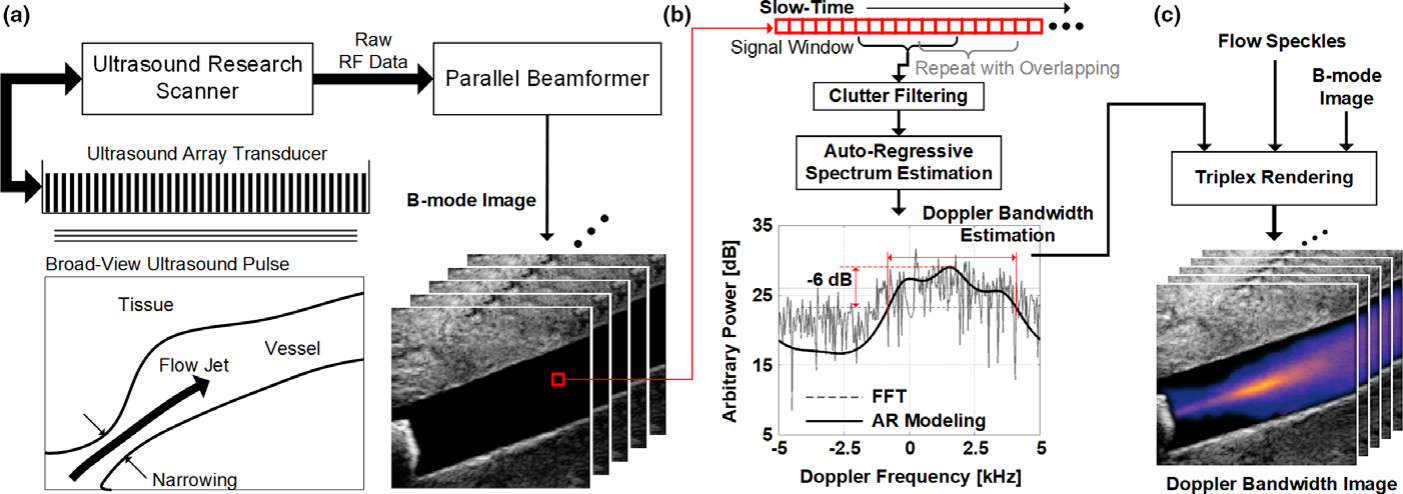
Conceptual illustration of DUBI. (a) Plane wave data acquisition at a region of interest using a research‐purpose ultrasound scanner that generates high‐frame‐rate ultrasound images; (b) Overview of the Doppler bandwidth estimator for a slow‐time ensemble extracted from the B‐mode images (indicated by the red box in (a)), with the estimated Doppler power spectrum (dashed line) and the AR estimated spectrum (solid line) shown for illustration; (c) Doppler bandwidth images (DUBI frames) generated by overlaying color‐coded Doppler bandwidth estimates on top of flow speckles and the B‐mode image. [Color figure can be viewed at wileyonlinelibrary.com]

As illustrated in Fig. 1(a), a plane wave excitation scheme was used to acquire slow‐time signals simultaneously over the entire image view. In line with our previously published work,^45^, ^46^ for each acquisition, an unfocused pulse was first transmitted through the array transducer to insonate the entire image view. On reception, raw radiofrequency (RF) data were acquired from each array channel and were beamformed in parallel to generate a full‐view image. This pulsing sequence was repeated to capture temporal information over different phases of a cardiac cycle. In turn, a 3‐D data matrix [Fig. 1(a) lower right corner] was formed by stacking the generated image frames. Clutter filtering was then applied to the data matrix along the slow‐time axis to suppress tissue echoes for every pixel [see Fig. 1(b)]. After that, Doppler bandwidth was estimated from each filtered blood flow signal ensemble as described in the next subsection.

#### 2.A.3. Doppler bandwidth estimation through autoregressive modeling of slow‐time signal

For a given pixel position, its Doppler bandwidth over a short period was estimated by first deriving an AR model of the slow‐time ensemble and then estimating the signal model's spectral power. In the DUBI framework, the Doppler bandwidth was determined as the full‐width at half maximum (FWHM) of the power spectrum. This process was repeated for every image pixel to generate a DUBI frame as a depiction of flow instability over the image view. To track the evolution of Doppler bandwidth over time, the entire estimation procedure was repeated at subsequent time points (*M* slow‐time samples apart) to generate a time series of DUBI frames over the image view, as illustrated in Fig. 1(b).

##### Derivation of AR model parameters

In the DUBI framework, the *n* ^th^ sample in a slow‐time ensemble with *N* samples was modeled by the following equation for a *P* ^th^‐order complex AR model representation^35^, ^36^:

(1) $$x\left[n\right]=-\sum_{k=1}^{P}a_{P,k}x\left[n-k\right]+e\left[n\right]$$

where *a*_*P,k*_ is the *k* ^th^ complex AR parameter of the model, and *e*[*n*] is the *n* ^th^ sample in the complex modeling error. The set of AR parameters {*a*_*P,k*_} was iteratively estimated using Burg's method^34^, ^47^ that yields better spectral estimation reliability.^48^ Burg's method works by iteratively minimizing the mean of forward and backward prediction errors.

##### Doppler bandwidth estimation approach

With the AR parameters, the modeled power spectrum of the slow‐time signal was next computed. For this task, Doppler power spectrum *S*_AR_[*f*] for the ensemble *x*[*n*] was constructed from the AR model through parametric spectral fitting as defined by the following equation:

(2) $$S_{\mathrm{AR}}\left[f\right]=\frac{\sigma_{p}^{2}\Delta t}{{\left|1+\sum_{k=1}^{P}a_{P,k}e^{-j2\pi fk\Delta t}\right|}^{2}}$$

where *f* is the bin frequency, Δ*t* is the pulse repetition interval and *σ*_*p*_^2^ is the average of mean powers of forward and backward prediction errors. After normalizing *S*_AR_[*f*] to its maximum power, the Doppler bandwidth of the slow‐time ensemble was determined as the FWHM of *S*_AR_[*f*], as illustrated in the spectrum shown in Fig. 1(b).

##### Advantages of AR‐based doppler bandwidth estimation

Through the use of AR modeling, DUBI's Doppler bandwidth estimation process became a task of finding the FWHM over a smoothened Doppler power spectrum. In turn, it was not susceptible to random spectral spikes that might arise in Doppler power spectra derived from the classical periodogram approach,^38^ thereby improving the consistency of Doppler bandwidth estimates. Such improvement in estimation performance has been confirmed by past investigations that used AR modeling in Doppler signal analysis.^37^ Another advantage of deriving the Doppler power spectrum using an AR modeling approach was that the spectrum could be reconstructed with a finer spectral resolution and not be bounded by the number of samples in the slow‐time signal. In doing so, the resulting Doppler bandwidth estimates were more accurate as they were less prone to discretization noise.

#### 2.A.4. DUBI as a triplex display mode

With the derived Doppler bandwidth estimates at every pixel position over different time instants, DUBI frames were formed as a new triplex display scheme to facilitate visualization of flow instability in an image view over time. As shown in Fig. 1(c), DUBI would synchronously display: (a) the Doppler bandwidth map annotating the flow instability of each pixel location; (b) flow speckle pattern revealing the flow trajectory; (c) B‐mode image showing the anatomical structure. Note that DUBI's flow speckle co‐visualization was adopted from our group's previous work on color‐encoded speckle imaging, in which flow speckle values were derived at different pixel positions by calculating the power of the corresponding slow‐time ensemble after clutter filtering.^45^ To form DUBI's triplex display, the flow speckle map was first overlaid on top of the B‐mode image at flow regions and was displayed as the base layer. Then, the Doppler bandwidth estimates were mapped to a thermal hue with brighter colors corresponding to higher Doppler bandwidths. This color map was subsequently overlaid using alpha compositing principles. For nonflow region, its transparency was set to 100% to reveal the anatomical structure. For flow regions, the transparency was set to 70% to reveal the flow speckle pattern and the color‐coded Doppler bandwidth estimates. This rendering strategy was repeated over different time points, and the image frames were stacked together to form a cineloop.

### 2.B. Implementation methods of DUBI

#### 2.B.1. Imaging hardware and data acquisition

DUBI was implemented on a programmable research platform that was built upon our group's prior work in ultrasound flow imaging innovations.^45^, ^46^ The platform consisted of a 128‐channel programmable transmit front‐end (SonixTouch; Analogic Ultrasound, Peabody, MA, USA), a pre‐beamformed DAQ tool with 40 MHz sampling rate and 12‐bit resolution,^41^ and a high‐speed processing platform based on graphics processing unit (GPU) technology (GTX 1080; NVidia Corporation, Santa Clara, CA, USA) for beamforming and signal processing. Our research platform was programmed to perform high‐frame‐rate data acquisition as required. Broad‐view acquisition at 10 kHz was achieved with unsteered plane wave excitation (0° transmission; 5 MHz center frequency, 5‐cycle pulse) using an L14‐5 linear array (0.3048 mm pitch; Analogic Ultrasound). Accordingly, raw data frames were acquired at a rate of 10,000 fps. Also, the transmit pulse shape yielded an effective axial range of 0.77 mm according to established formulas.^49^ In each acquisition, the raw channel‐domain data were stored on the DAQ tool until the internal 16 GB memory buffer was filled (3 s duration at 5 cm imaging depth). The data were then streamed offline to the GPU platform for processing.

#### 2.B.2. Plane wave image formation

For each frame of the acquired dataset, parallel beamforming was performed on the GPU computing platform using a codec programmed in Matlab (R2016a; Mathworks Inc., Natick, MA, USA) in which the GPU‐accelerated parallel beamforming library^50^ was invoked. The codec first applied a 3–7 MHz bandpass filter to the received RF data on a per‐channel basis to suppress out‐of‐band white noise. The filter was implemented as a finite‐impulse‐response (FIR) filter in Matlab, with minimum filter order (30 taps) formulated using the Parks‐McClellan equiripple design algorithm.^51^ The analytic form of the acquired data was subsequently obtained using a FIR‐based Hilbert transformer (50th order) as described earlier.^50^ With the analytic RF data, image frames (with 0.2 mm pixel spacing) were finally parallel beamformed using our GPU‐based delay‐and‐sum algorithm (64 array channels were used with Hanning apodization). This three‐stage process was repeated for the data of different slow‐time sampling instants, thereby generating a stack of image frames over slow‐time for Doppler signal processing and bandwidth estimation. Note that, for our 64‐channel receive aperture configuration, the effective lateral width was estimated to range between 0.31 and 0.77 mm for a 2–5 cm imaging depth range, as determined based on well‐known formulas.^49^ Also, with its apodization profile, our receive beamformer's maximum sidelobe magnitude (occurred at 2 cm depth) was found to be 27.6 dB lower than that for the main lobe, according to in‐house point target simulations.

#### 2.B.3. Signal processing for doppler bandwidth estimation

Doppler bandwidth estimation was performed at various slow‐time instants on a per pixel basis. First, a Doppler clutter filter was applied to suppress tissue echoes; this filter was implemented as a FIR high‐pass filter with 0.05 normalized cutoff frequency (i.e., 250 Hz for 10 kHz slow‐time sampling rate), and its filter order was optimized to be 135 taps using the equiripple filter design algorithm. For flow regions, Doppler bandwidth estimation was performed over each slow‐time ensemble with N* *=* *100 samples, equivalent to windows of 10 ms observation period. This relatively short duration was chosen to limit spectral broadening induced by rapid acceleration (and deceleration) of blood flow. The corresponding AR‐based Doppler spectrum was subsequently derived as described in Section 2.B. For our implementation, an 8th‐order AR model was chosen as its performance has been shown to be similar to higher order models (up to 16th order) in earlier work.^37^ Also, the AR‐modeled power spectrum was formed with a 10 Hz spectral resolution (i.e., 0.001 normalized frequency relative to slow‐time sampling rate) to avoid spectral discretization noise.

To accelerate the derivation of each DUBI frame, Doppler bandwidth estimation for multiple pixels was executed concurrently by devising a GPU‐based parallel computing kernel for AR modeling. This GPU kernel was implemented using the C++ programming language and the compute unified device architecture application programming interface (ver. 7.5; NVidia Corporation). Its formulation, as explained in the Appendix, was based on a public‐domain computing algorithm for Burg's method.^52^ After completing the Doppler bandwidth estimation process for each DUBI frame, it was repeated at other slow‐time sampling instants to generate a time series of Doppler bandwidth maps. In particular, a repetition was performed after shifting the observation window by 25 samples along slow‐time (i.e., M* *=* *25; with 75% overlap for N =* *100). The effective frame rate of the Doppler bandwidth maps was 400 fps (10,000 fps raw data frame rate divided by 25). The resultant Doppler bandwidth maps were finally rendered as described in Section 2.A.4.

### 2.C. Experimental testing methods

#### 2.C.1. Nozzle‐based unstable flow model

To evaluate the performance of DUBI in identifying unstable flow zones, a nozzle‐flow setup was devised to generate flow conditions ranging from laminar to turbulent flow. The flow conditions were characterized by their Reynolds number *Re*, defined as *Re *= *uD/ν*, where *u* is the average flow velocity, *D* is the nozzle diameter and *ν* is the fluid kinematic viscosity. Since the average velocity term *u* is known to be equal to flow rate *Q* divided by cross‐sectional area *A* (i.e., *u *= *Q*/*A*), the Reynolds number could be readily rewritten as *Re *= 4*Q/πDν*. Based on this relation, we realized different values of *Re* by changing the flow rate. In turn, a series of flow conditions with progressing degree of flow disturbance was generated by increasing the flow rate. For each of these flow conditions, the stable and unstable flow regions were identified with the aid of CEUS (to be discussed in Section 2.C.3).

**Figure 2:**
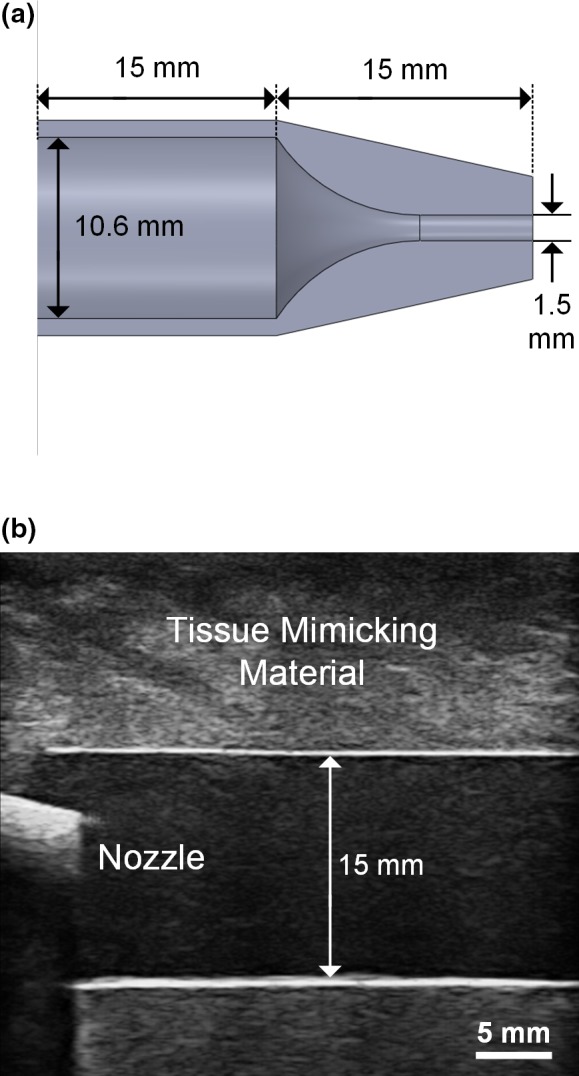
Overview of nozzle‐flow phantom. (a) Cross‐sectional view of the discharge nozzle showing the narrowing flow channel; (b) B‐mode image of the discharge nozzle inserted into a 15 mm diameter wall‐less PVA flow phantom (the interior of the discharge nozzle cannot be visualized due to acoustic shadowing of the fabrication material). [Color figure can be viewed at wileyonlinelibrary.com]

In terms of the nozzle design, it was shaped as a curved funnel to progressively narrow the flow channel's diameter from 10.6 to 1.5 mm over a 15 mm passage [see Fig. 2(a)] which gradually increased the flow velocity.^53^ The model's base was elongated by 15 mm so that a flow connector (EW‐06361‐61; Cole‐Parmer, Vernon Hills, IL, USA) could be securely affixed to the base end. The nozzle was then inserted into a phantom made of poly‐vinyl alcohol (PVA) (fabrication details discussed in Section 2.C.2) for flow to be discharged into a 15 mm diameter straight tube flow channel. The phantom provided an acoustic window to image the discharged flow from the nozzle. Note that this nozzle‐flow geometry serves as an idealized model of stenosis (with 90% reduction in diameter in this case).

#### 2.C.2. Fabrication of nozzle‐flow model and flow circuit setup

3‐D printing was leveraged to physically construct the nozzle. First, its physical dimensions were drafted on computer‐aided design (CAD) software (SolidWorks; Dassault Systems, Waltham, MA, USA). To compile 3‐D printing instructions, the CAD model (saved in STL stererolithography file format) was imported into a slicer software (KISSlicer; ver 1.5). The instructions were then downloaded to a fused deposition modeling (FDM) system (Model DX; CreatBot 3D Printer, ZhengZhou, China) with a nozzle size of 0.25 mm to create the physical models. Layer and skin thicknesses of 0.1 and 0.5 mm were used, respectively.

The flow phantom was fabricated based on investment casting principles according to the protocol reported in our previous work.^54^ In this study, a PVA‐based wall‐less flow phantom with a 15 mm diameter flow channel across a tissue mimicking slab was fabricated. The flow channel was formed by first embedding a straight rod in PVA solution and then removing the rod after the solution has congealed. The straight rod was 15 mm in diameter with a length of 280 mm, and it functioned as the negative replica of the flow channel. For the phantom to grip onto the inlet nozzle and outlet flow connector, 30 mm on both ends of the rod were narrowed to 9 mm diameter (effective length of the flow channel became 220 mm). Similar to the nozzle, the inner core was drafted on SolidWorks and was physically constructed using the FDM system with the same settings. Next, the rod was gently polished using an abrasive paper (400 grit size) and was suspended in the phantom case (80 × 295 × 70 mm^3^, w × l × h) by mounting it onto two side plates. Tissue mimicking material was casted around the straight rod by: (a) pouring PVA solution into the phantom case, (b) administering three freeze–thaw cycles (freeze in −20°C for 24 h followed by thawing at 4°C for 24 h). Note that the PVA mixture consisted of 10% PVA (341584; Sigma‐Aldrich, St Louis, MO, USA), 3% graphite (282863; Sigma‐Aldrich), 0.3% potassium sorbate (85520; Sigma‐Aldrich), and 86.7% distilled water. The acoustic attenuation and speed of this tissue mimicking material were respectively 0.229 dB/(cm·MHz) and 1535 m/s as reported earlier.^54^ Upon completing the thermal cycling process, the flow channel was instated by simply sliding the straight rod out from one end of the phantom.

After the flow phantom was fabricated, the nozzle was affixed into the inlet flow connector. The setup was then connected to a programmable flow pump (details described elsewhere^52^) that fed blood mimicking fluid at constant flow rates according to the parameters listed in Table 1 to generate a range of flow conditions as discussed earlier. Note that the blood mimicking fluid was fabricated using an Orgasol‐based standardized formula^55^ and a laboratory procedure that we have described previously.^54^ Its dynamic viscosity (4.1 mPa∙s) and density (1037 kg/m^3^) were matched to that for human blood. Figure 2(b) shows a B‐mode image of the inlet segment of the assembled flow phantom captured using a clinical scanner (SonixTouch; Analogic Ultrasound).

**Table 1:** Flow rate and ROI sizes for each flow condition

Flow conditions in reynolds number *Re*	Flow rate (ml/s)	ROI length (mm)	
		Stable	Unstable
375	1.8	34.1	–
750	3.5	18.2	8.4
1125	5.2	6.2	19.2

#### 2.C.3. Identification of unstable flow region with CEUS

To facilitate the identification of regions with the presence of unstable flow, microbubble contrast agents were administered to trace its flow trajectories.^56^ The rationale behind was that laminar flow occurs when fluid flows in parallel layers with no disruption between the layers. As such, microbubbles in stable regions would move in a straight path. On the contrary, unstable flow would correspond to cases where trajectories of the microbubbles were observed to have crossed each other's path. In accordance with this notion, a bolus of microbubble contrast agents (USphere Prime; Trust BioSonics, Hsinchu, Taiwan) were slowly injected manually to the inlet of the flow phantom for CEUS imaging.

Plane wave data acquisitions were repeated for all flow rates using the same data acquisition scheme described in Section 2.B.1, but with a 5 MHz, 2‐cycle pulse at 50% of the original transmit power instead to excite the microbubbles in the stable cavitation regime. High‐frame‐rate CEUS images were then generated through the same image formation method described in Section 2.B.2. To highlight flow trajectories, high‐persistence B‐mode images were rendered by averaging the beamformed RF signal magnitude over multiple frames before log compression. This allowed the hyperechogenic microbubbles to create streaks along their trajectory, representing the flow path lines. This process was repeated every *K* frames with overlapping to generate a cineloop of flow path lines for analysis.

The image persistence and overlapping (i.e., effective frame rate) were adjusted according to the flow rate to normalize the microbubbles’ retention time (in terms of frame numbers) and in turn generate a consistent trace. Table 2 summarizes the CEUS‐rendering parameters for three representative flow conditions. Regions where microbubbles were observed to cross path were identified and classified as unstable flow region. On the other hand, regions before the laminar flow layers broke down were categorized to be in stable flow condition. Intermediate boundaries where flow transitioned from laminar to turbulent were also identified for all flow conditions. Regions of interest (ROIs) were selected manually using a Matlab built‐in function; the height of the ROI was set to 1.5 mm to match the nozzle diameter. The length of ROIs varied depending on the position of the intermediate boundaries; a 2.5 mm margin was reserved for both stable and unstable zones from the intermediate boundaries as a conservative stance in avoiding ambiguity when selecting ROIs for performance analysis. ROI sizes of each zone for the different flow rates are summarized in the two rightmost columns of Table 1.

**Table 2:** Contrast‐enhanced ultrasound cineloop rendering parameters

Flow conditions in reynolds number *Re*	Persistence *K* (Frames)	Overlapping (Frames)	Effective frame rate (fps)
375	240	224	625
750	120	112	1250
1125	80	74	1667

#### 2.C.4. ROC analysis of doppler bandwidth measurements

To assess DUBI's sensitivity and specificity in determining flow instability, an ROC analysis was conducted. The procedure involved the following key steps. First, the measured Doppler bandwidth estimates within the ROIs for all image frames were classified as either belonging to the stable flow (negative) or unstable flow (positive) groups based on the CEUS reference data. Next, a bandwidth threshold was set to categorize the measured bandwidths to their predicted conditions (stable or unstable); Doppler bandwidth above the threshold was categorized as positive (i.e., unstable) and vice versa. True negative (TN) and false positive (FP) were computed from the stable group, while true positive (TP) and false negative (FN) were counted from the unstable group. This process was repeated at different bandwidth thresholds ranging from 0.1 to 10 kHz with 0.1 kHz increment, and each corresponding set of TN, FN, TP, and FP values was computed. Using these data, the sensitivity [*TP*/(*TP*+*FN*)] and specificity [*TN*/(*TN*+*FP*)] of the test were derived to plot the ROC curve. The area under curve was calculated as a summative measure of the ROC. Also, Youden index (*Sensitivity*+*Specificity*–1) was computed for all points on the ROC curve to identify the optimal cutoff that maximizes both sensitivity and specificity with equal weight.^57^

#### 2.C.5. Comparative analysis with CFI

The performance of DUBI was contrasted against that for conventional CFI. To facilitate such comparison, CFI frames were computed by applying CFI's scanline‐based imaging paradigm to re‐process the raw channel‐domain datasets that were acquired as described in Section 2.B.1. Specifically, our platform's GPU beamformer was reconfigured to perform quad‐line parallel receive beamforming on each frame of channel‐domain data. The full image view was divided into 48 zones, each of which comprised four beams in adjacent lateral positions. Quad‐line beamforming was performed over each zone for 10 consecutive pulsing events before advancing to the next zone. Accordingly, at each pixel position in a CFI scanline, the slow‐time ensemble was 10 samples in size with 10 kHz sampling rate, yielding an observation period of 10 ms (i.e., same as that for DUBI). The effective CFI frame rate was 20.8 fps. For each slow‐time ensemble, tissue clutter was suppressed using a first‐order infinite impulse response high‐pass filter (0.05 normalized cutoff; with projection initialization),^58^ and then mean flow velocity and velocity variance were estimated via Kasai's autocorrelation algorithm.^59^ The flow estimates of different pixels in the CFI frame were mapped to a hot‐cold hue to render mean flow velocity information. They were also mapped to a tricolor hue to render both mean velocity and variance information, in line with previously published work.^29^, ^30^, ^31^

#### 2.C.6. Case demonstration using anthropomorphic phantoms

To further demonstrate the efficacy of DUBI to detect flow instability in a physiologically relevant condition, a series of imaging experiments was conducted on a healthy carotid bifurcation model, a moderately stenosed bifurcation (50% eccentric stenosis relative to the internal carotid artery diameter, as defined based on the NASCET criterion^60^), and a severely stenosed bifurcation (75% eccentric stenosis). These geometries have well‐studied flow characteristics as obtained from Doppler ultrasound^15^ and particle image velocimetry.^16^

The bifurcation phantoms were fabricated using the same investment casting procedures as described in Section 4.B. The vessel cores (healthy, 50% and 75% eccentric stenosis) were identical to the core geometries previously reported,^61^ for which the unstenosed diameters of the common, internal, and external carotid artery branches were 6.0, 4.2, and 3.5 mm, respectively. The vessel cores were first drafted using CAD software (SolidWorks) and were physically fabricated using the FDM system mentioned earlier. The physical builds of the vessel cores were subsequently embedded in PVA solution inside a phantom box (80 × 295 × 70 mm^3^, w × l × h) and three freeze–thaw cycles were administered to solidify the PVA solution. Lastly, the vessel geometries were instated by removing the core (through snapping the core at the bifurcation site and sliding out the snapped parts from both ends).

During experiments, the bifurcation phantoms were connected to the programmable flow pump that was driving a pulsatile flow profile (20 ml/s systolic flow rate; 60 bpm). Plane wave imaging was performed with the transducer surface angled at 20° against the phantom surface using a custom‐made PVA coupling wedge. Note that our use of the angled coupling wedge was inspired by another study that used slanted gel pads to generate more favorable beam‐flow angles when performing clinical Doppler ultrasound.^62^ With this experimental configuration, DUBI cineloops were obtained using the same protocol as described in previous subsections.

## 3 Results

### 3.A. Findings from nozzle‐flow phantom

#### 3.A.1. DUBI revealed spatiotemporal dynamics of unstable flow patterns

Doppler ultrasound bandwidth imaging was found to be effective in visualizing the spatiotemporal dynamics of unstable flow patterns (in both magnitude and spatial distribution) that cannot be perceived with conventional Doppler ultrasound. Movie S1 shows DUBI cineloops of three flow conditions with *Re* of 375 (left column), 750 (center column) ,and 1125 (right column). Note that the beam‐vessel angle was 70° in all three cases. Cineloops on the top row of Movie S1 shows high‐frame‐rate DUBI (400 fps effective frame rate) being played back in slow motion at 50 fps. CFI cineloops are shown in the middle row for mean flow velocity mapping at a frame rate of 20.8 fps, and in the bottom row for co‐rendering of mean velocity and velocity variance (referred to as Doppler variance maps in prior work^29^, ^30^, ^31^).

As shown in Movie S1, for both DUBI and CFI (velocity maps and Doppler variance maps), stable laminar flow stream at low *Re* can be visualized, and transitional flow from laminar to unstable disturbance at higher *Re* can be observed. However, since CFI velocity maps after all renders estimated mean flow velocity, it is difficult to use this imaging mode to visualize where flow instability started to develop at higher *Re* values. CFI variance maps were also not effective in highlighting the starting point of flow instability in the nozzle‐flow phantom. On the other hand, for DUBI, its measured Doppler bandwidths were observed to be commensurate with the increase in *Re*. One particular observation is that the high Doppler bandwidth pattern (bright orange color) started to diverge after passing through the mid‐section of the imaging view. This trend enabled us to use DUBI to more effectively identify the development of flow instability.

#### 3.A.2. DUBI was effective in depicting unstable flow regions

**Figure 3:**
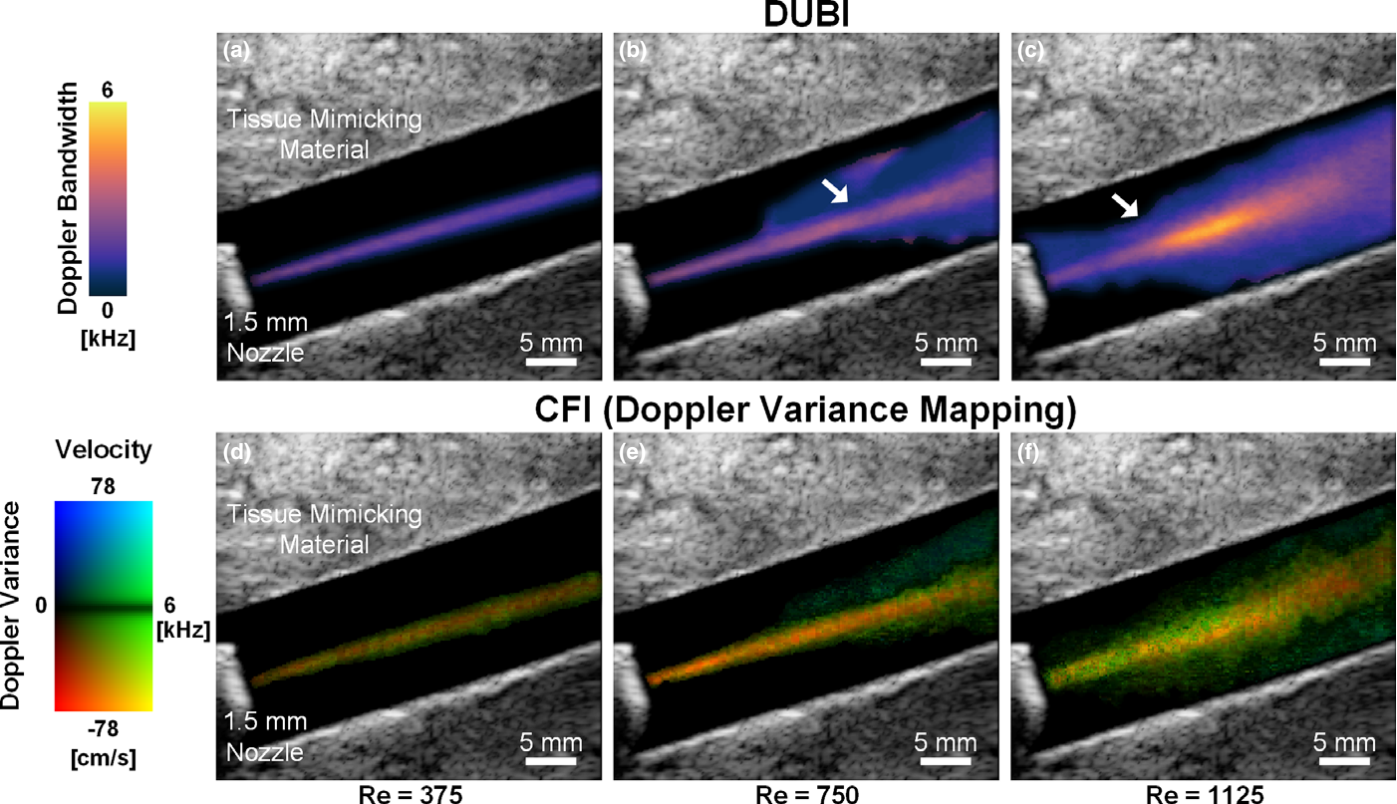
Maps of mean Doppler bandwidth (top row; derived from DUBI) and mean Doppler variance (bottom row; derived from CFI variance mapping) acquired over a 0.5‐s period in a nozzle‐flow phantom. Results for three Re values are shown: 375 (left column), 750 (middle column), and 1125 (right column). The white arrows indicate the intermediate boundaries between stable flow (narrow flow stream) and unstable flow (diverging pattern). [Color figure can be viewed at wileyonlinelibrary.com]

To further analyze the trends observed in Movie S1, still frames of mean Doppler bandwidth were obtained by averaging over 0.5 s for the three flow conditions. Results are shown in Fig. 3 (top row) with the intermediate boundary between stable and unstable flow indicated by white arrows (as determined from CEUS). Comparative findings derived from CFI variance mapping (i.e., mean Doppler variance) are also shown on the bottom row of Fig. 3. One general observation to be noted is that, as *Re* increased from 375 to 750 and 1125, the maximum value in DUBI's mean Doppler bandwidth maps had increased from 2.1 to 3.1 and 5.2 kHz. More importantly, for the *Re* = 1125 case, the development of unstable flow corresponded to a spatial peak zone in the mean Doppler bandwidth maps derived from DUBI. The 5.2 kHz spatial peak value in the unstable flow zone was significantly higher than the spatial maximum of 2.4 Hz in the upstream flow jet near the nozzle. Such visualization was not clearly highlighted in the mean Doppler variance maps, because the upstream flow jet was found to yield similar mean Doppler variance values (spatial maximum: 6.7 kHz) as those in the unstable flow region (spatial maximum: 7.1 kHz).

#### 3.A.3. DUBI yielded similar findings as CEUS

To confirm that pixel positions with high Doppler bandwidth estimates in DUBI correspond to unstable flow, CEUS cineloops acquired from the three identical flow conditions are rendered as Movie S2 for comparison with Movie S1. As illustrated in Movie S2, trailing path lines of contrast agents were highlighted by high‐persistence rendering of Movie S2. As shown in this movie, microbubbles moved in straight path lines when the flow rate (and *Re*) was low (as highlighted in the left cineloop); correspondingly a low Doppler bandwidth was recorded in Movie S1.

In contrast, as *Re* increased (*Re* = 750 and 1125), streak traces of contrast agents crossing paths were evident (center and right cineloops of Movie S2). They signify flow perturbations in the form of the mixing of flow layers. These flow perturbations became more significant as they propagated and eventually transitioned to unstable flow. Positions where flow path lines deviated from the straight trajectory were also reflected by the high Doppler bandwidth observed in Movie S1, as well as Fig. 3(b) and 3(c).

**Figure 4:**
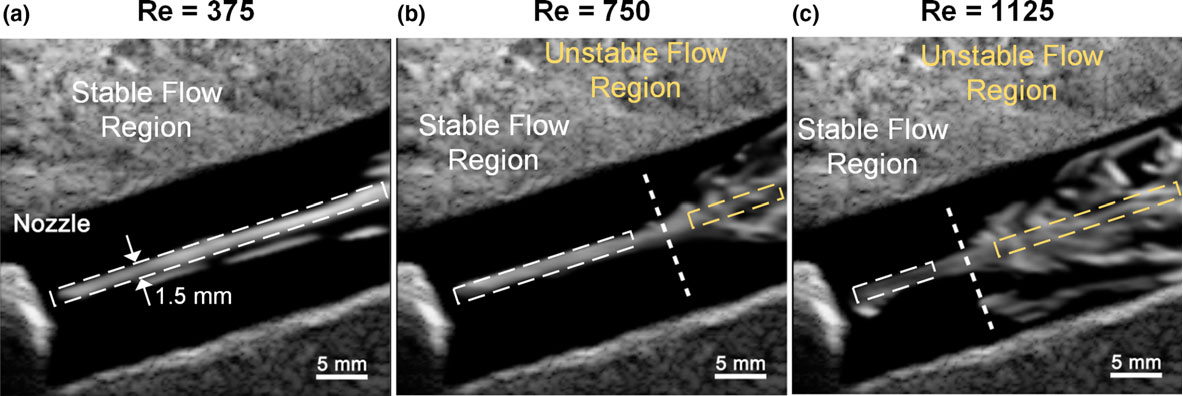
Representative CEUS image frames obtained from (a) *Re *= 375 with persistence = 240 frames, (b) *Re *= 750 with persistence = 120 frames, and (c) *Re *= 1125 with persistence = 80 frames. Thick dash lines identify the intermediate boundaries; flow traces were straight before the dash line but began to diverge afterwards. The white and yellow boxes (1.5 mm in height) respectively indicate the stable and unstable flow region used for ROC analysis. [Color figure can be viewed at wileyonlinelibrary.com]

As a further analysis, Fig. 4 shows selected CEUS frames of Movie S2 with dashed lines drawn on the figure to indicate the intermediate boundary between stable and unstable flow. The location of these boundaries in the high *Re* cases (middle and right frames) was found to be in close proximity with the location where a rise in Doppler bandwidth estimate started to appear in Fig. 3, thereby indicating that Doppler bandwidth can be a reliable indicator to discern flow instability.

#### 3.A.4. DUBI showed strong ROC performance in mapping flow instability

**Figure 5:**
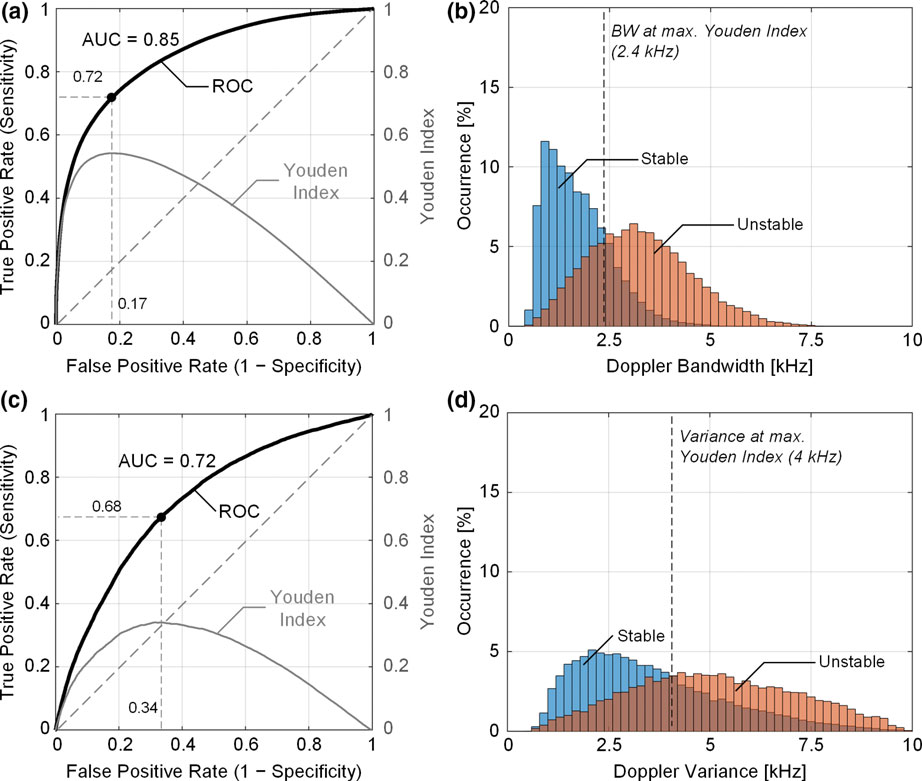
Sensitivity and specificity analysis of DUBI. (a) ROC curve (dark line) for unstable flow classification (with an area under curve of 0.85) and the corresponding Youden index (gray line) at different false positive rates; the black dot indicates the optimal classification performance when Youden index attained its maximum. The diagonal dash line represents the theoretical ROC curve for random guessing. (b) Doppler bandwidth distributions from stable (in blue) and unstable flow (in orange) regions, with the optimal bandwidth threshold marked by the black dash line. The deeper color depicts the overlapping area between the two distributions. Corresponding result for Doppler variance mapping are shown in (c) and (d) for comparison. The area under ROC curve for Doppler variance mapping was 0.72. [Color figure can be viewed at wileyonlinelibrary.com]

Figure 5(a) plots the ROC curve (dark line) of DUBI with samples collected from all the ROIs at the three flow rates. As can be observed, the ROC curve (with an area under curve of 0.85) was positioned above the diagonal line (gray dashed line), thereby indicating that DUBI has positive predictive power in determining flow instability. The Youden index is also plotted in Fig. 5(a) (gray line) as a global indicator of sensitivity and specificity. The optimal Youden index was found to be 0.54 when sensitivity and specificity were respectively 0.72 and 0.83. This optimal point corresponded to a bandwidth threshold of 2.4 kHz, as indicated in the bi‐population histogram shown in Fig. 5(b). These ROC findings represent significant improvements over the ones obtained from Doppler variance mapping. As shown in Fig. 5(c) and 5(d), the optimal sensitivity and specificity of Doppler variance mapping were 0.68 and 0.66, and they were achieved with a maximum Youden index of 0.34 and a bandwidth threshold of 4.0 kHz. The area under ROC curve for Doppler variance mapping (0.72) was also lower than that for DUBI.

### 3.B. Findings from carotid bifurcation experiments

#### 3.B.1. DUBI highlighted unstable flow emerging from stenosis site

When DUBI was applied to pulsatile carotid bifurcation phantoms, it was found to capable of highlighting flow instability that arises downstream from the stenosis site in diseased bifurcations. The top row of Movie S3 shows a series of DUBI cineloops for different bifurcation geometries: healthy (left) and diseased (center: moderate 50% eccentric stenosis; right: severe 75% eccentric stenosis). For reference, the bottom row of Movie S3 shows the corresponding Doppler spectrograms taken from a 1 × 1 mm^2^ sample volume placed at the flow jet, as indicated in the cineloop.

The primary observation to be noted is that the range of Doppler bandwidths increased in the stenosed vessel (upper branch) because of the lumen narrowing at the stenosis site. This observation was consistent with our findings from the flow nozzle model, whereby an increasing *Re* would result in greater Doppler bandwidth (in the bifurcation experiments, the flow profile remained unchanged but the “nozzles” were narrower as stenosis increased). Doppler bandwidths in the healthy model were low (<1.4 kHz) throughout the entire cardiac cycle. In contrast, high Doppler bandwidths (>2.4 kHz) were observed in both diseased models, especially at the flow jet region during flow systole and the dicrotic wave phase of the cardiac cycle.

**Figure 6:**
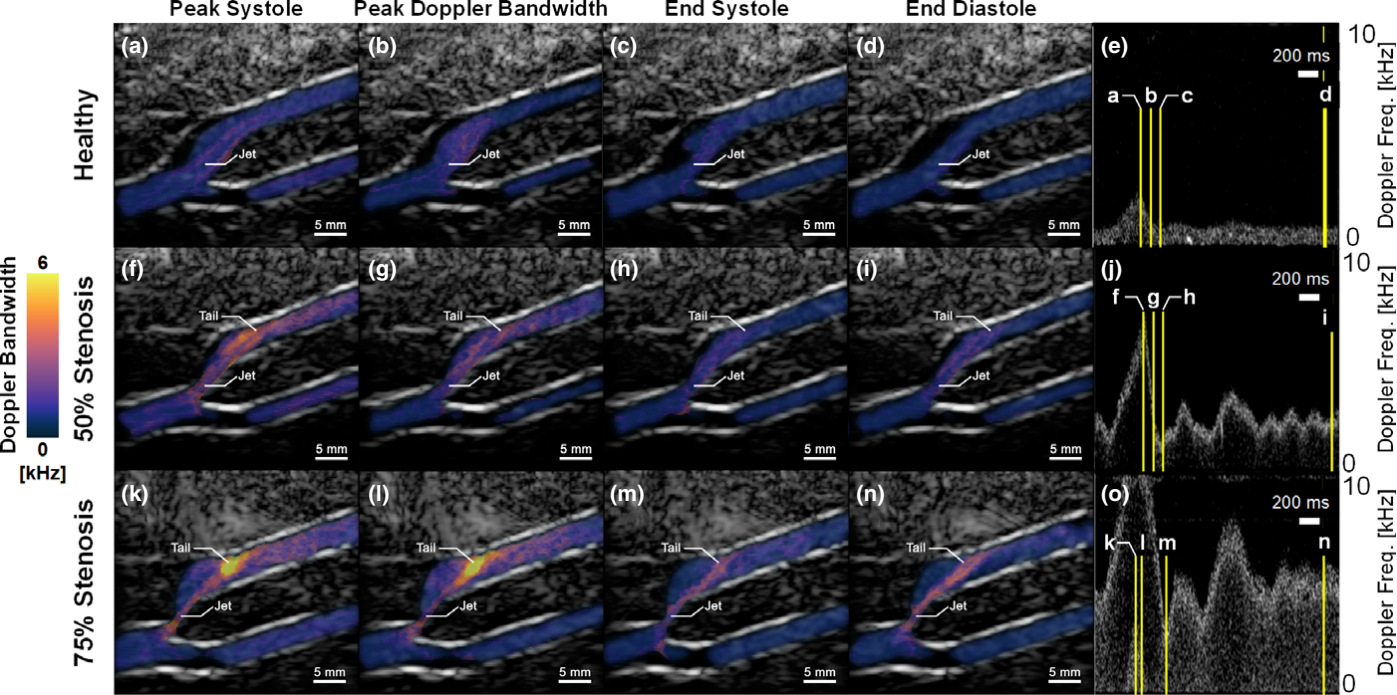
DUBI frames at different phases of the cardiac cycle. Top, middle, and bottom rows respectively show Doppler bandwidth maps measured from (a–d) healthy, (f–i) 50% eccentric stenosis and (k–n) 75% eccentric stenosis bifurcation phantoms with their corresponding Doppler spectrogram shown on the rightmost column (e, j, o). First to fourth columns show frames captured at (a, f, k) peak systole, (b, g, l) peak flow disturbance, (c, h, m) end systole and (d, i, n) end diastole respectively with their relative time points indicated on the spectrograms. [Color figure can be viewed at wileyonlinelibrary.com]

Selected frames of DUBI are shown in Fig. 6 to facilitate further interpretation of the information provided by DUBI at specific time points of interest. This figure depicts frames from peak systole (column 1), at the instant with peak Doppler bandwidth measured in a cardiac cycle (column 2), end systole (column 3), end diastole (column 4), and the corresponding Doppler spectrogram at the stenosis site (column 5). Three main observations can be made. First, the maximum Doppler bandwidth increased as the degree of stenosis increased, as reflected by the brighter thermal hue in DUBI frames. Second, the peak Doppler bandwidth was found at the jet tails [Fig. 6(k) and 6(l)] where flow perturbations were strongest. Third, for the 50% stenosis model (Fig. 6 middle row), significant increase in Doppler bandwidth was only found during flow systole, whereas for the 75% stenosis model, its high‐range Doppler bandwidth sustained throughout the entire cardiac cycle (Fig. 6 bottom row). The timing and positions of peak Doppler bandwidth were in general consistent with those measured using turbulence intensity under similar flow conditions as previously reported.^15^, ^16^

#### 3.B.2. Maximum doppler bandwidth is correlated with degree of stenosis

**Figure 7:**
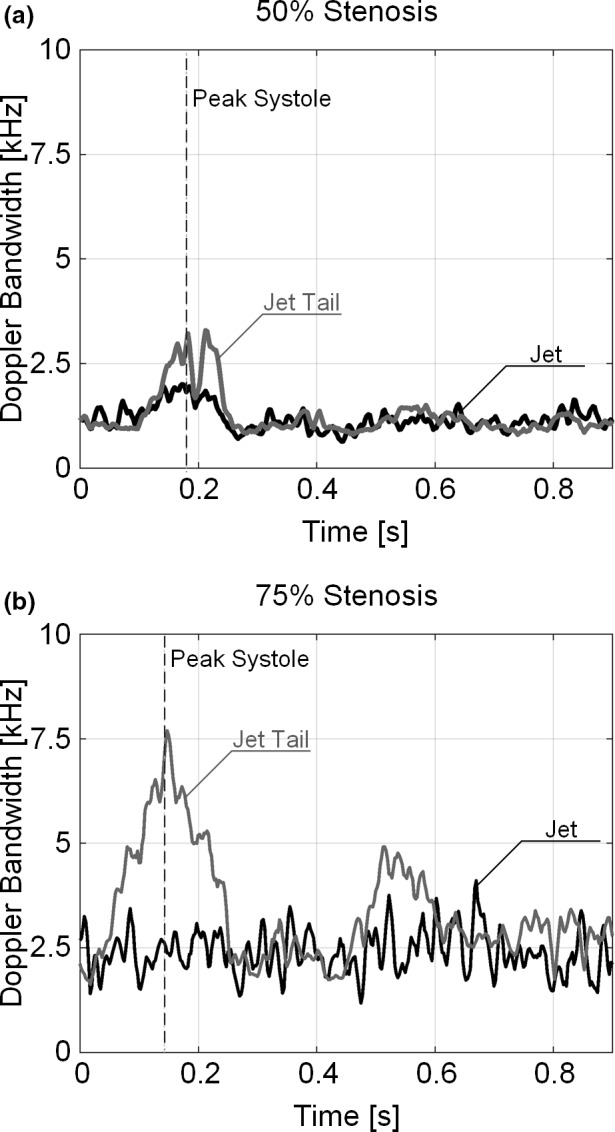
Time traces of Doppler bandwidth acquired at the jet (dark line) and the jet tail (gray line) as marked in Movie S3 for (a) 50% and (b) 75% stenosed bifurcation. The gray dotted line marks the time point at peak systole.

As a further analysis of DUBI, Fig. 7 shows time traces of the measured Doppler bandwidths at the stenotic jet area (dark line) and its tail (gray line) for the two diseased bifurcation phantoms. A dash line to indicate peak systole is also included in this figure, and the sample volume positions are labeled in Movie S3. For the 75% stenosis model, the maximum Doppler bandwidth at the jet tail was significantly higher (7.7 kHz) compared to that for the 50% stenosis model (3.3 kHz). Also, the Doppler bandwidth at the jet tail in the 75% stenosis model shows greater temporal fluctuation over the cardiac cycle, and it expectedly showed a decreasing trend during end systole (100 ms after peak systole) since flow deceleration naturally favored reestablishment of stable flow conditions. Another point worth noting is that in both diseased models, the Doppler bandwidth at the flow jet was lower than that at the jet tail. This finding expectedly indicates that flow instability mainly emerged not at the flow jet, but downstream from the jet. For the 75% stenosis model, at the stenotic flow jet, Doppler bandwidth was found to show a greater extent of fluctuation. This trend is likely because the higher temporal variation in jet speed for the 75% stenosis model naturally favors transitioning between stable and unstable flow regimes.

## 4 Discussion

### 4.A. DUBI as a new framework for mapping flow instability

Visualizing unstable flow noninvasively is not a trivial task. In particular, two practical flow characteristics must be addressed when devising a new flow instability mapping framework: (a) at a given time instant, unstable flow pattern may vary spatially because of its dissipative nature; (b) over a cardiac cycle, flow conditions may vary temporally due to the pulsatile nature of blood flow. DUBI has been specifically designed to visualize and track these spatiotemporal dynamics. It is equipped with three key features that have collectively enabled visualization of unstable flow. First, it can track spatial variations in flow instability (via local Doppler bandwidth measurements) over the entire image view at high‐frame‐rates beyond the video display range [Fig. 1(a)]. Second, it uses an AR modeling approach to consistently derive Doppler bandwidth estimates [Fig. 1(b)]. Third, its triplex display approach enables simultaneous visualization of flow instability information (Doppler bandwidth), flow trajectory (flow speckles), and the anatomical background [Fig. 1(c)].

We have demonstrated DUBI's efficacy in identifying unstable flow for a series of flow conditions ranging from laminar to turbulent flow. The performance of DUBI was first evaluated on a nozzle‐flow setup (Fig. 2) with CEUS images acquired as benchmarking references (Movie S2 and Fig. 4). Unstable flow regions were found to correspond to high Doppler bandwidth regions in DUBI (Movie S1 and Fig. 3). Such correspondence was broadly found to be sensitive and specific in comparison to conventional CFI mapping of Doppler variance (Fig. 5). The practical merit of DUBI was also established through a series of carotid bifurcation experiments (Movie S3 and Fig. 6). It was found to be effective in identifying unstable flow at the jet tail downstream from a stenosis site (Fig. 7).

DUBI represents the first image‐based, noninvasive flow instability mapping framework with fine temporal resolution. From a clinical diagnosis standpoint, this framework unlocks new potentials in improving atherosclerotic disease management. For example, emergence of unstable flow can indicate the onset of plaque formation, so DUBI may help facilitate early diagnosis of atherosclerosis. In addition, new insights on plaque progression may be obtained in correlation with the intensity and size of flow instability zones, since unstable flow has been shown to contribute to the progression of an atherosclerotic plaque.^8^ Moreover, given that our nozzle‐flow setup has demonstrated initial potential in detecting flow instability emerging from a stenosed flow outlet, DUBI may be further developed as a new tool in valvular stenosis diagnostics to complement other emerging ultrasound techniques.^63^

### 4.B. Limitations and future work

In the future, DUBI can be further refined to address a few technical shortcomings. First, since the Doppler equation is well known to be dependent on the beam‐flow angle, the Doppler bandwidth estimates are also inherently dependent on this parameter. It would be beneficial to derive angle‐independent Doppler bandwidth estimates by developing a data regularization scheme based on the local beam‐flow angle at each pixel position, which can be estimated using a flow vector estimator.^64^, ^65^ Note that the regularization scheme should also take into account the issue of intrinsic spectral broadening due to the finite transit time of blood scatterers through the sample volume (the extent of intrinsic spectral broadening is known to be angle‐dependent as well).^44^ In doing so, the sensitivity and specificity of DUBI can likely be further improved.

From a signal processing standpoint, two enhancements can be made to improve the performance of DUBI in the presence of tissue motion. First, adaptive clutter filtering techniques like eigen‐filtering can be introduced to improve the quality of flow detection.^66^, ^67^ The real‐time feasibility of this technique has already been established,^68^ and its statistical performance has been confirmed to be superior to conventional clutter filters.^69^ Second, in making Doppler bandwidth measurements, it may be worthwhile to introduce the use of more robust model‐based estimators like the Matrix Pencil technique^70^ that derives coefficients via total‐least‐squares error minimization instead of AR modeling's least‐squares error minimization. Such improvement will increase the consistency of the resulting Doppler bandwidth estimates.

## 5 Conclusion

Flow instability is undisputedly a significant biomechanical factor that influences the pathophysiology of atherosclerosis. In this paper, we presented a new high‐frame‐rate ultrasound framework called DUBI to noninvasively map flow instability over an image view at fine temporal resolution by tracking the local Doppler bandwidth over time. With this technique, new functional indices for unstable flow quantification may be derived in the future. In turn, this framework can potentially help facilitate risk stratification of atherosclerotic plaques with better efficacy than existing routines used in clinical practice.

## Supporting information


**Movie S1:** Cineloops of DUBI and CFI for different values of *Re* in the nozzle‐flow phantom as presented in Sec. 3.A.1.

**Movie S2:** CEUS cineloops of various nozzle‐flow conditions for comparison with Movie S1. See Sec. 3.A.3 for interpretation.

**Movie S3:** DUBI cineloops for three carotid bifurcation phantom geometries as described in Sec. 3.B.1.

## Appendix 1 GPU‐based implementation of Burg's method

### 1.1

For our GPU computing kernel for Doppler bandwidth estimation, a thread block was allocated to handle the estimation of Doppler bandwidth for the slow‐time ensemble of one pixel in the DUBI frame. Note that the corresponding slow‐time ensemble *x*[*n*] was first transferred to the shared memory of the GPU for fast data access. The thread block, containing *N* threads (i.e., same as the slow‐time ensemble size), then proceeded to derive the set of *P*th‐order AR parameter values for that slow‐time ensemble by executing a fast implementation of Burg's method^50^ that involved *P* iterations from *p *=* *1 to *p *= *P*. Within each iteration, two computational steps were carried out. First, the compute threads were tasked to calculate the forward and backward prediction error ensembles, respectively denoted as $e_{p}^{\;f}$[*n*] and $e_{p}^{b}$[*n*]. Specifically, for the *p*th iteration, the *n*th thread was tasked to compute the *n*th sample in the following error ensemble (each with *N* samples):

(A1) $$e_{p}^{\;f}\left[n\right]=x\left[n\right]+\sum_{k=1}^{p}a_{p,k}x\left[n-k\right]$$

(A2) $$e_{p}^{b}\left[n\right]=x\left[n\right]+\sum_{k=1}^{p}a_{p,k}x\left[n-p+k\right]$$

Note that the mean of (A1) and (A2) corresponded to the error term *e*[*n*] in the AR model stated in (1) for the *p*th iteration.

In the second computational step of the same iteration, a sub group of threads were tasked to compute the intermediate AR parameters *a*_*p,k*_. It achieved so by updating the intermediate AR parameters using the following equations:

(A3) $$a_{p,i}=a_{p-1,i}+\varphi_{p}a_{p-1,p-i}^{*}$$

where *i *=* *1, 2, …, *p*–1, *a*_0,0_ = 1, * denotes complex conjugate, and

(A4) $$a_{p,p}=\varphi_{p}$$

(A5) $$\varphi_{p}=\frac{-2\sum_{n=p}^{N-1}e_{p-1}^{\;f}\left[n\right]e_{p-1}^{b*}\left[n-1\right]}{\sum_{n=p}^{N-1}\left({\left|e_{p-1}^{\;f}\left[n-1\right]\right|}^{2}+{\left|e_{p-1}^{b}\left[n-1\right]\right|}^{2}\right)}$$

The set of AR model coefficients was finalized after repeating the above computational steps for *P* iterations.
